# Comparison of the Inguinal and High Scrotal Approaches for the Treatment of Inguinal Hernias in Boys

**DOI:** 10.4314/ejhs.v31i4.11

**Published:** 2021-07

**Authors:** Adebayo Gbenga Tanimola, Ademola Olusegun Talabi, Oludayo Adedapo Sowande, Olusanya Adejuyigbe

**Affiliations:** 1 Division of Paediatric Surgery, Department of surgery, Obafemi Awolowo University/Obafemi Awolowo University Teaching Hospitals Complex, Ile-Ife, Osun State, Nigeria

**Keywords:** High scrotal, conventional, inguinal hernia, boys

## Abstract

**Background:**

The conventional groin incision herniotomy is still being adhered to despite high success rate of high scrotal approach. Hence, the aim of this study was to compare the outcomes of high scrotal and conventional approaches for the treatment of inguinal hernia in boys.

**Methods:**

A prospective study of 100 boys with 108 inguinal hernias whose ages were less than 15 years. They were randomized into 2 groups; high scrotal and conventional approaches. Ninety-four patients with 101 hernias were analyzed. The operative time, conversion rate (high scrotal approach), and postoperative complications were reported.

**Results:**

A total of 100 boys with 108 hernias were enrolled but 94 patients with 101 hernias were analyzed. They comprised of 48 patients with 51 hernias in the high scrotal group and 46 patients with 50 hernias in the conventional group. Their age range was between 2 months and 168 months with a mean of 47.9 ± 46.7 months. The conversion rate of high scrotal approach was 1.9%. The mean duration of operation in the high scrotal group was 37.1 ± 13.3 minutes compared with 37.2 ± 15.1 minutes in the conventional group, p = 0.982. Early postoperative scrotal edema was more in the high scrotal group compared to the conventional group, p = 0.018. The Hollander wound evaluation score was better in the high scrotal incision compared to the conventional approach, p = 0.003.

**Conclusion:**

The high scrotal approach may be an alternative to conventional herniotomy in boys.

## Introduction

Paediatric inguinal hernias result from the persistence or failure of involution of the processus vaginalis. Males are more commonly affected than females in a ratio of 3–10:1. It is commoner on the right side than the left side and is bilateral in 5–10% of cases. Most surgeons advocate early diagnosis and prompt surgical treatment in order to avoid complications such as strangulation obstruction and testicular atrophy (1,2).

The mainstay of treatment of paediatric inguinal hernia is herniotomy. The basic principle of open herniotomy involves identifying the spermatic cord and vessels, dissection of the processus vaginalis and its subsequent high ligation. Inguinal hernia repair in children has evolved from open- to minimal access- surgery in the last three decades (3–6). However, this technique is yet to be fully deployed in our environment due to high cost of set-up and steep learning curves (7). The conventional groin skin crease incision technique leaves attendant complications which include wound and scrotal haematoma (leading to compression of the vas deferens), vassal injury (micro infracts), iatrogenic undescended testis, wound infection and ugly scar (5,8,9). These morbidities may have future adverse effect on fertility and the psychology of these young patients (1,10).

The high scrotal incision approach which was originally described by Bianchi and Squire (11) in 1989, for the management of palpable undescended testis lying distal to the external inguinal ring has been adopted by many researchers as an alternative to the conventional method in managing inguinal hernias in children. The technique provides direct access to the spermatic cord and the external inguinal ring with less dissection and disruption of tissues in the groin (8,12). This technique has been described as well tolerated, cosmetically pleasing with adequate maintenance of the testis in the scrotum by many investigators in Turkey (12), Malta (13) and Denver, Colorado, USA (14).

There are few reports documenting the suitability of high scrotal incision in boys in our environment. This study thus sought to compare the early outcomes of high scrotal approach with conventional groin skin crease incision in the surgical treatment of indirect inguinal hernias in boys less than 15 years in a Nigerian tertiary hospital.

## Materials and Methods

This was a prospective randomized study conducted at the paediatric surgical unit of a tertiary teaching hospital in Southwest Nigeria over a period of 3 years between May 2015 and April 2018. The hospital offers primary, secondary and tertiary health care services to a populace who are predominantly farmers, artisans and civil servants. The participants were boys whose ages were less than 15 years. Inclusion criteria were uncomplicated inguinal hernias and children whose parents gave consent to participate in the study. Exclusion criterion was previous groin surgery. Participants were given full right to withdraw from the study at any time. Consent to conduct the study was obtained from the Ethics and Research Committee of our institution. The demographic characteristics, side(s) of the groin involved, early post-operative complications (scrotal edema and haematoma, pain requiring analgesia within 24 hours of operation) and cosmetic appearance of the scar on postoperative day 30 were recorded in a spreadsheet. Late complications such as iatrogenic undescended testis, testicular atrophy and recurrent inguinal hernia were documented. Other information collected were the educational levels of parents and their phone contacts.

**Fig. 1:**
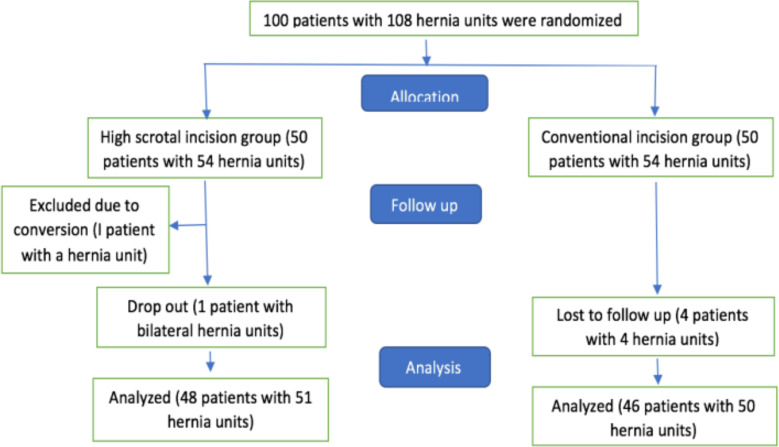
Disposition of patients assigned to the study

Patients were randomized into two groups, conventional groin crease (GC) incision and high scrotal incision (HS) groups. One-hundred envelopes, each containing a small piece of paper marked A (GC, n = 50) or B (HS, n = 50) placed in a bag were picked by balloting just before induction of general anesthesia. Where a child has bilateral hernia, each side was randomized separately. Patient(s) that required conversion were mentioned but excluded from analysis. Patients who dropped out or lost to follow up were also excluded from analysis (Figure 1). Though 100 patients with 108 inguinal hernias were enrolled into the study, only 94 patients with 101 hernias were analyzed. All patients were fasted for a minimum of 4 hours. They were started on intravenous fluids as they arrived in the theater. None of the patient had perioperative antibiotics.

The procedures were all done as day case surgery. All operations were performed under general anaesthesia. The duration of operation was measured from the commencement of skin incision to skin coverage with the aid of stop watch by an anaesthetist assistant. All the surgeries were performed by a single surgeon. All patients were reviewed on postoperative day 4, 7, 14 and 30 for early wound complications such as haematoma, oedema and discharges. The high scrotal incision technique: Patient was placed supine on the operation table and general anaesthesia was applied using face mask. Skin preparation was done using combination of cetrimide (3%) and chlorhexidine (0.3%), and methylated spirit over the groin and the perineum. Sterile drapes were applied to isolate the operative field.

**Figure 2:**
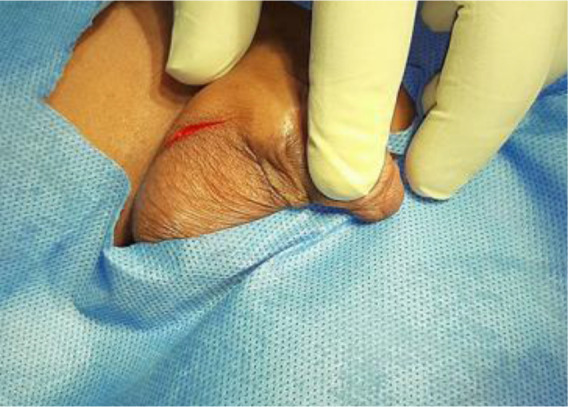
A curvilinear incision of about 2cm to 3cm long over the upper scrotal ruggae

A transverse incision of about 2cm to 3cm was made on the upper scrotal crease (Figure 2), and deepened to the subcutaneous layer which was divided with the dissecting scissors while holding the edges of the incision high using toothed forceps to avoid injury to the spermatic cord structures. Through a combination of blunt and sharp dissection, the spermatic cord was visualized and delivered into the wound. Haemostasis was maintained with electrocautery. The cord coverings were divided in order to identify the vas deferens, testicular vessels and the hernia sac. The hernia sac was dissected away from the vas and vessels.

**Figure 3:**
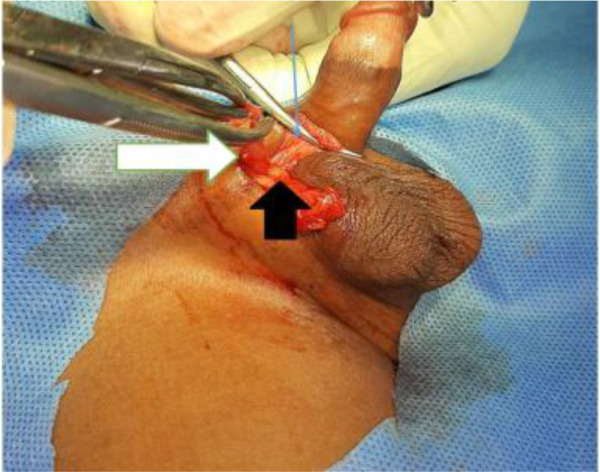
The dissection, division and high ligation of the hernia sac from the spermatic cord via high scrotal approach. The white arrow depicts the hernia sac, thin blue arrow represents the spermatic fascia while the short black arrow shows the cord structures (vas deferens and testicular vessels)

**Figure 4:**
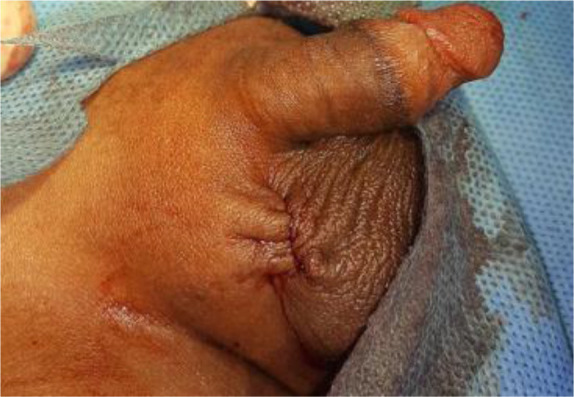
Wound closure of high scrotal herniotomy.

**Figure 5:**
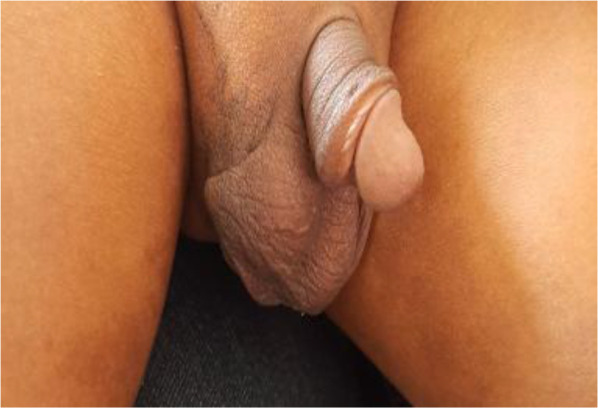
The postoperative scrotal scar

A retractor was placed within the wound to retract the skin toward the superficial inguinal ring and in line with the inguinal ligament. Blunt dissection was continued until the neck of the sac was seen at the level of the internal ring and preperitoneal fat. The sac was then transfixed and ligated using 3/0 Vicryl (polyglactin) suture (Figure 3). Vas deferens and vessels were approximated by downward traction on the testicle and haemostasis was checked. The dartos muscle were approximated by few interrupted 3/0 Vicryl inverted stitches. Skin was closed by a subcuticular suture using Monocyrlrapide (Figure 4). The cosmetic appearance of wound scar on postoperative day 30 is shown in Figure 5.

The conventional approach group (GC) was as described for the traditional transverse groin crease incision in children. The parental level of satisfaction with the cosmetic appearance of wound scars were assessed using visual analogue scale while the Hollander wound evaluation score (15) was used by a surgeon who did not participate in the study to assess the cosmetic appearance of scars in both groups on postoperative day 30. We follow - up the participants for 36 months to observe presence of late postoperative complications such as iatrogenic undescended testis, testicular atrophy and recurrence of inguinal hernia. We defined uncomplicated inguinal hernia as a hernia with no symptoms and signs of incarceration, intestinal obstruction and strangulation. We also defined early postoperative complications as those occurring within 30 days of operation. Those who developed postoperative complications were managed appropriately.

Data were presented as frequencies, means and standard deviation for continuous variables. Student's t-test was used to test the significant differences between mea values. Chi-square was used to test the significant differences between categorical variables. The level of significance was taken as p <0.05.

## Results

A total of 100 boys with 108 hernias were enrolled into the study but 94 patients with 101 hernia units were analyzed. They consisted of 48 patients with 51 hernias in the high scrotal group and 46 patients with 50 hernias in the conventional group. Their ages ranged between 2 months and 168 months with a mean of 47.9 ± 46.7 months. The age of those in the high scrotal group was 2 months to 168 months with a mean of 46.6 ± 50.2 months while in the conventional group, the age range was 2 months to 156 months with a mean of 46.7 ± 43.1 months. There was no statistically significant difference in the mean age of patients in both groups, t = -0.231, p = 0.818 as shown in Table 1. Eighty-seven (92.6%) patients had unilateral hernias consisting of 57 (65.5%) boys with unilateral right inguinal hernias and 30 (34.5%) boys with unilateral left inguinal hernias. Seven (7.4%) patients had bilateral inguinal hernias (Table 1).

**Table 1:** Demographic characteristic, side of hernia, duration of operation and wound outcomes

Variables	Techniques		
	
	High scrotal (HS)	Groin crease (GC)	P value
Age range (months)	2 – 168	2 – 156	
Mean age (months)	46.6 ± 50.2	46.7 ± 43.1	0.818
Side of hernia			
Unilateral	45	42	
Bilateral	3	4	
Duration of operation			
Range (minutes)	14 – 75	15 – 86	
Mean (minutes)	37.1 ± 13.3	37.2 ± 15.1	0.982
Complications (on postoperative day 4)			
Scrotal haematoma	3 (5.9%)	2 (4.0%)	1.000
Scrotal edema	22 (43.1%)	10 (20%)	0.018
Mean Hollander wound evaluation score	5.84 ± 0.36	5.50 ± 0.71	0.003

The only converted case (n = 1 hernia unit, 1.9%) in the high scrotal approach was a 12-year old boy with right inguinal hernia. The reason being that there was difficulty in identifying the hernia sac (bubonocele) which was high up in the inguinal canal. The remaining hernia (processus vaginalis) units were successfully ligated, giving a success rate of 98.1%. The conventional cases were all successfully ligated high up in the groin (100%).

The duration of operation in the high scrotal group was between 14 to 75 minutes with a mean of 37.1 ± 13.3 minutes whereas in the conventional group, it was 15 to 86 minutes with a mean of 37.2 ± 15.1 minutes. The difference was not statistically significant, t = 0.025, p = 0.982 as highlighted in Table 1.

No patient had pain requiring additional analgesia within 24 hours after surgery in both study groups. Transient scrotal haematoma was observed in 3 wounds in the high scrotal group compared to 2 scrotal haematoma in the conventional group on the fourth post-operative day visit. The difference was not statistically significant, p = 1.000. However, when seen on the 7 post-operative day, all scrotal haematoma had resolved. Scrotal edema was found in 22 (43.1%) repairs in the high scrotal group compared to 10 (20%) in the conventional group on the 4^th^ post-operative day visit. The difference was statistically significant, p = 0.018 as shown in Table 1. They were subsequently placed on conservative therapy by simple elevation of the scrotum. On the 7^th^ post-operative day, scrotal edema had resolved in some of the herniotomies leaving 10 (19.6%) cases in the high scrotal group and 7 (14%) cases in the conventional group. However, when seen on the 14^th^ post-operative visit, the remaining scrotal edema had resolved. All wounds healed satisfactorily without infection in both groups.

The mean Hollander wound evaluation score was 5.84 ± 0.36 in the high scrotal group and 5.50 ± 0.71 in the conventional group. The difference was statistically significant, t = -0.069, p = 0.003, (Table 1). The result of the parental level of satisfaction with the cosmetic appearance of surgical scar is as shown in Table 2. There was no statistical significant difference in the parental satisfaction with the surgical scar in both groups, p = 0.596. In this study, no iatrogenic undescended testes, testicular atrophy were seen (n = 0, 0%) during 36 months of follow up. Also there was no recurrence of inguinal hernia in any patient (n = 0, 0%) during this period.

**Table 2:** Parental satisfaction with scar appearance

Technique	Level of assessment			
	
	Fair	Good	Excellent	P-value
	N (%)	N (%)	N (%)	
High scrotal	0 (0.0)	19 (37.3)	32 (52.7)	0.364
Conventional	1 (2.0)	23 (46.0)	26 (52.0)	

## Discussion

The high scrotal approach lends itself to repair of groin pathologies, especially inguinal hernias, hydroceles and undescended testes in boys (3,4,13,16). Indeed, this technique provides a direct access to the processus vaginalis, external inguinal ring and inguinal canal up to the deep ring due to the relative mobility of skin in this region of the body in children (12, 14, 17, 18). In the present study, the success rate of high scrotal herniotomy of 98.1% was comparable to that of the conventional approach (100%) and this is in tandem with a comparative study by Gokcora et al (8). Bahaaeld in et al in a study of 104 herniotomies in 96 boys using scrotal crease incision observed a comparable success rate of 100% (9). Other studies comparing the success rates of high scrotal method with conventional approach on the repair of congenital hydrocele in children found similar success rates (3, 4). This implies that the high scrotal incision may be an effective alternative to the conventional method in children with minimal tissue dissection and preservation of the anatomical integrity of the inguinal canal.

Previous studies (18,19) documented that the operating time using high scrotal technique was shorter than that of conventional technique. In this study, the operating timein both approaches were comparable. In a study by Bahaaeldin et al (9), they found a mean operating time of 22.40 ± 4.139 minutes using high scrotal incision herniotomy which was lower than the 37.1 ± 13.3 minutes documented in our study. We attributed the difference in the operating time to the slow learning curve of the surgeon in our study even though the technique had been deployed for palpable undescended testes in our practice (17). We intend to deploy this technique routinely for common groin pathologies (hydrocele, undescended testis and inguinal hermia) in boys in our hospital.

We had a conversion rate of 1.9% in the high scrotal method approach. This was in a 12 years' old whose hernia sac was high (bubonocele) in the inguinal canal. It was difficult to identify and ligate the sac from a lower incision. Bahaaeldin et al (9) in their series encountered difficulty during the procedure to ligate the processus vaginalis in the inguinal canal in 5 children. However, they there was no conversion in their series. We believe that a child with a bubonocele-type of inguinal hernia should be recognized *ab initio* (clinically before surgery) so that the hernia sac is not missed during single incision technique. Failure to identify the hernia sac may lead to recurrence of inguinal hernia on the long term.

The major recognizable complications of high scrotal incision are wound infection, scrotal edema and or haematoma. In this series we had no incidence of wound infection as the wounds healed satisfactorily (11). However, a significant number of patients in the high scrotal group had scrotal edema in the early post-operative period compared to the conventional approach. This edema subsided gradually and disappeared within 14 days on conservative therapy. We attribute the difference in the rate of scrotal edema to excessive retraction of the scrotal skin leading to disruption of the cuticular lymphatics draining the scrotum. Gokcora et al (8) in a prospective comparative study observed 12 cases of scrotal haematoma – edema in the transverse high scrotal incision group compared to 10 cases of scrotal haematoma – edema in the conventional group. Iyer et al (16) in their series of 563 scrotal incisions for groin pathologies in boys reported 2 cases of scrotal haematoma and 2 scrotal swelling and pyrexia. Also in this study, we had no cases of recurrence of inguinal hernia and no patient presented with testicular atrophy or iatrogenic undescended testes. This is consistent with other studies (3, 9).

A lesser postoperative pain is the major catalyst for early researchers to propagate the use of high scrotal incisions for groin pathologies in children (11, 20, 21). In our study, there were no patients that required additional pain medications in the first 24 hours after surgery in both groups contrary to these reports. This may be due to the subjective interpretation of what constitute pain by the parents of these children in our study. This constitute a limitation to this study. Indeed, a more objective tool should have been used to assess postoperative pain in this study. However, this will require admitting patients overnight which may negate the principle of day case surgeries for minor elective procedures. The cosmetic appearance of the wound is one of the main advantages of the high scrotal approach over the traditional groin crease incision. Bianchi and Squire as well as other researchers (8, 11, 18, 19) highlighted the fact that the high scrotal approach offers excellent cosmetic appearance as the scar is hidden within the scrotal ruggae. In this study, the parental assessment of the surgical scars was similar in both groups. This may also be due to the subjective interpretations of the parents of children in this study. However, we found that the cosmetic appearance of scars using Hollander wound evaluation score was better in the high scrotal group compared to the conventional method. This finding is supported by the observations of other researchers (17, 20, 21).

In conclusion, the high scrotal incision compares favourably with conventional approach in terms of successful high ligation and excision of the hernia sac and operative time. The cosmetic appearance of wound scar using Hollander wound evaluation score appear to be better in the high scrotal incision compared to convention groin incision herniotomy. We recommend that the high scrotal approach may be an option to consider during herniotomy in children.
